# Flood vulnerability level analysis as a hydrological disaster mitigation effort in Krueng Jreue Sub-Watershed, Aceh Besar, Indonesia

**DOI:** 10.4102/jamba.v11i1.737

**Published:** 2019-09-12

**Authors:** Helmi Helmi, Hairul Basri, Sufardi Sufardi, Helmi Helmi

**Affiliations:** 1 Doctoral Study Program of Agriculture Sciences, Universitas Syiah Kuala, Darussalam, Banda Aceh, Indonesia; 2 School of Forestry Science, Teungku Chik Pante Kulu, Banda Aceh, Indonesia; 3 Faculty of Agriculture, Universitas Syiah Kuala, Darussalam, Banda Aceh, Indonesia

**Keywords:** land use, flood vulnerability, flood zone map, hydrological disaster mitigation, Sub-Watershed Krueng Jreue

## Abstract

The flood phenomenon in the Krueng Jreue Sub-Watershed, Aceh Besar, Indonesia, in recent years indicates biophysical damage to the land. Floods are influenced by factors from biophysical conditions of the land and high rainfall with small river cross-sectional capacity causing water to overflow the embankment and flood low areas. This research aims to analyse the flood vulnerability level in the Krueng Jreue Sub-Watershed, Aceh Besar, Indonesia. The results showed that flood vulnerability in the research area consisted of four classes: very vulnerable, vulnerable, moderately vulnerable and somewhat vulnerable, with each area averaging a score of 43.0, 38.8, 30.0 and 21.7. Types of land use that are particularly vulnerable to flooding are rice fields with a mean total score of 43.0. The vulnerable classes are found in settlements and moorings, with a total score of 42.0 and 36.5, respectively. While open land, shrubs, grasslands, primary forests and secondary forests are quite vulnerable to flooding, with a mean total score of 32.5 each: 30.0, 30.0, 28.0 and 27.0. The main components affecting flood vulnerability are rainfall, temperature and land use, while additional components are soil infiltration and slope. Mechanised hydrological disaster mitigation can be performed through optimisation of weir, embung, rorak and check-dam. Vegetative hydrological mitigation efforts can be performed by reforestation and agroforestry systems, maps and flood prediction. Non-technically, hydrological disaster mitigation efforts can be undertaken with legal policies, law enforcement, map creation and prediction of droughts and socialisation of legislation.

## Introduction

Land and water resources are related to the hydrologic cycle. Climate change has an effect on changes in the hydrological cycle, such as floods and droughts as hydrological disasters. A flood phenomenon is dominated by high rainfall and an overflow of river water (Andriyani et al. 2010). Flooding from day to day is widespread and routinely happens in every rainy season. The scope of the area widened from the usual areas affected by floods to the surrounding area. Delays in handling flooding are generally because of information about the biophysical condition of a watershed. For flood mitigation, a mapping of vulnerable watersheds and flood risk is required.

Hydrological disaster cannot be avoided but can be anticipated with the development of science and technology supported by accurate data. Early warning as non-structural measures implemented in developing countries (Jayawardena 2015) is a major factor in disaster risk reduction. Such action is necessary to anticipate the occurrence of a hydrological disaster so that losses caused by the disaster can be minimised.

This fact suggests the importance of understanding the characteristics of the region and its response to changes in the hydrological cycle resulting from climate change (Van Huijgevoort et al. 2014). An understanding of the Krueng Jreue Sub-Watershed provides important information in planning, area management and early anticipation of the negative impact and risk of damage caused by a hydrological disaster, both in the short and long term. Based on these problems, it is necessary that research aims to analyse the causes of hydrological disasters that occur in the sub-watershed based on biophysical and climatological aspects. Through this research, hydrological disaster mitigation efforts in the Krueng Jreue Sub-Watershed was obtained, and the negative impact and risk of flood damage can be minimised.

## Methods

This research uses descriptive method, field survey and laboratory analysis. Observation of monthly rainfall data for the 2005–2014 period were obtained from the Indrapuri station. In determining the level of flood vulnerability, this research uses quantitative analysis and the results of calculations of flood vulnerability parameters including rainfall, land use, soil infiltration and slope followed by weighting, blooming and the score to get flood vulnerability levels. The weighting of each parameter is shown in Tables 1–4.

**TABLE 1:** Rainfall classification.

No.	Rainfall (mm year^−1^)	Description	Weight	Level	Score
1	> 3.000	Height	-	5	5
2	2.500–3.000	Rather high	-	4	4
3	2.000–2.500	Medium	1	3	3
4	1.500–2.000	Rather low	-	2	2
5	< 1.500	Low	-	1	1

*Source*: Pusat Penelitian Tanah dan Agroklimat, 1995, *Laporan Akhir. Database Iklim dan Sistem Informasi Iklim*, Balitbang Pertanian, Bogor.

**TABLE 2:** Classification of land use.

No.	Land use	Weight	Level	Score
1	Open land, rivers, reservoirs, swamps, pasture	-	5	10
2	Settlements, mixed garden	-	4	8
3	Agriculture, paddy fields, mooring	2	3	6
4	Plantations, shrubs	-	2	4
5	Primary forest, secondary forest	-	1	2

*Source*: Meijerink, A.M.J., 1970, *Photo-interpretation in hydrology: A geomorphological approach*, Enschede Netherlands: International Institute for Aerial Survey and Earth Sciences, p. 142.

**TABLE 3:** Soil infiltration classification.

No.	Soil texture†	Infiltration rate‡	Weight	Level	Score
1	Clay	Very slow	-	5	15
2	Clay sandy	-	-	-	-
	Clay dusty	-	-	-	-
	Latex clay	Slow	-	4	12
3	Clay sandy Clayed	-	-	-	-
	Clay dusty	-	3	-	-
	Clay	Medium	-	3	9
4	Dusty clay	-	-	-	-
	Sandy clay	Fast	-	2	6
5	Sand	Very sand	-	1	3
	Clay sand	-	-	-	-

*Source*:

† Rahayu, S., Widodo, R.H., Noordwijk, V.N., Suryadi, I. & Verbist, B., 2009, *Monitoring air di DAS*, p. 104, World Agroforestry Center-Southeast Asia Regional Office, Bogor and

‡ Budianto, P.T.H., Wirosoedarmo, R. & Suharto, B., 2014, ‘Perbedaan laju infiltrasi pada lahan hutan tanaman industri pinus, jati dan mahoni’, *Jurnal Sumberdaya Alam dan Lingkungan* 1(1), 15–24.

**TABLE 4:** Slope classification.

No.	Gradient class (%)	Description	Weight	Level	Score
1	0 ≤ 8	Flat	-	5	20
2	8 ≤ 15	Sloping	-	4	16
3	15 ≤ 25	Somewhat steep	4	3	12
4	25 ≤ 40	Steep	-	2	8
5	≥ 40	Very steep	-	1	4

*Source*: Pusat Penelitian Tanah dan Agroklimat, 1995, *Laporan Akhir. Database Iklim dan Sistem Informasi Iklim*, Balitbang Pertanian, Bogor.

The flood vulnerability score based on the scores consists of five classes as listed in Table 5, that is, (1) very vulnerable, (2) vulnerable, (3) fairly vulnerable (moderate), (4) somewhat vulnerable and (5) not vulnerable.

**TABLE 5:** Flood vulnerability rate by score.

No.	Score	Flood rate vulnerability
1	42–50	Very vulnerable
2	34–41	Vulnerable
3	26–33	Fairly vulnerable (moderate)
4	18–25	Somewhat vulnerable
5	10–17	Not vulnerable

*Source*: Adapted from from Sigit, A.A., Priyono, P.P. & Andriyani, A.A., 2011, *Aplikasi SIG berbasis Web untuk monitoring banjir di wilayah DAS Bengawan Solo Hulu*, pp. 1–10, Seminar Nasional Teknologi Informasi & Komunikasi Terapan, Surakarta.

### Ethical considerations

This article followed all ethical standards for research without direct contact with human or animal subjects.

## Result and discussion

### Flood vulnerability based on land biophysical conditions

Flooding is caused by a number of factors that interact with each other so that it is very difficult to explain changes in flood hazard (Johnson et al. 2016). There are four variables determining the level of flood vulnerability based on the biophysical aspects of the land, namely: dynamic factors (rainfall and land use) and static factors (soil infiltration and slope). Seyhan (1995) states that the causes of flooding include (1) meteorological factors, namely rainfall conditions consisting of the amount, intensity and distribution; (2) watershed characteristics related to land cover conditions, topography, soil, geology and drainage density; and (3) human factors related to hydraulic structures, agricultural engineering and urbanisation.

Determination of natural factors (rainfall, soil infiltration and slope) and the management factor (land use) of the Krueng Jreue Sub-Watershed is based on the most dominant variables in the region. The provision of grading each type of variable that causes flooding uses the provision that the smaller the value given, the better the level of vulnerability.

### Rainfall variables

Rainfall is a major factor in controlling the hydrological cycle of a watershed area. The size of the water resources in a watershed depends on the amount of rainfall that occurs along the watershed. The rainfall used in the mapping analysis of the flood vulnerability zone is the average for 10 years (2005–2014) which is an average of 102.6 mm month^−1^. The area of a watershed that has high rainfall shows that the area has a high potential for flooding. If there is no rain falling on the earth’s surface, there will be no flooding in an area. The higher the intensity of rainfall, the more it will be vulnerable to flood disasters. The greater the intensity of rainfall, the greater the incidence of landslides (Sedogo 2002). The assessment of the effect of rainfall variables in the Krueng Jreue Sub-Watershed was given a weight of 1, while the value of the rainfall was dominated by a value of 2, at 95%. The highest score is 3, while the lowest score is 2 and the average is 2.0.

The greater the rainfall, the more vulnerable the area is to flooding, where rainfall is a dynamic factor compared to soil infiltration and slope. The high rainfall and the magnitude of the surface flow coefficient increasingly spurred areas prone to flooding (Verrina, Anugrah & Sarino 2013). The maximum water potential of surface flow from 70% – 75% rainfall becomes surface flow and 25% – 30% experiences infiltration and percolation (Nugroho 2002).

### Land use variables

Land use is one of the important factors in determining the level of flood vulnerability. The effect of land use on floods is very high. The lower the land cover, the more vulnerable to flooding (Utomo & Supriharjo 2012). Land use changes cause sub-watershed discharge and volume. Typically erroneous management without regard to environmental aspects results in disasters.

The assessment of the influence of land use variables in the Krueng Jreue Sub-Watershed was given a weight of 2. Most of the land use was secondary forests, shrubs and grasslands. Harkat is characterised by the value of 10 (33%). The highest score is 10, the lowest score is 2 and the average is 6.0. The lowest score is found in primary forest and secondary forest. Dense land cover occurs in the forest. Surface flow is lessened because of the role of canopy interception and the increasing rate of infiltration because of the high absorption capacity of litter (Wibowo, Hendro & Danuarti 2008).

According to Nugroho (2002), forest areas can drain 10% – 40% of rainwater so that it can absorb 60% – 90% of the rainwater, but if it becomes a settlement, the forest area will drain 40% – 75% of rainwater and absorb 25% – 60% of rainwater. The run-off coefficient (*C*) in large-value settlements (0.25–0.75) makes it difficult for water to seep into the soil (Biswas & Mandal 2014), causing a greater likelihood of flooding. Efforts to minimise run-off and low run-off coefficients are assets in the efficiency of water resources management (Savenije 1996), so that the flood disaster is reduced.

### Land infiltration variables

Infiltration is the flow of water into the soil as a result of gravity and capillary force. Variable land infiltration in determining the level of flood vulnerability is a reflection of whether or not rainfall permeates into the soil and the condition of soil texture (Anna, Suharjo & Priyana 2015). The more dense and low the absorption capacity of the soil against water, the more susceptible the land will be affected by floods. The rate of soil infiltration can be determined by the soil texture approach. The more rough the soil texture, the faster the rate of infiltration. This is because surface run-off water easily seeps into the soil and the possibility of flooding is low (Haghnazari, Shahgholi & Feiz 2015).

An assessment of the effect of soil infiltration variables in the Krueng Jreue Sub-Watershed was given a weighting of 3. Most soil infiltrations were categorised as very slow and slow, each with clay texture classes, spun clay and dusty clay. Harkat is dominated by grades 4 and 3 at 38%. The highest score is 15, the lowest score is 3 and the average score is 10.6.

Soil texture affects the rate of infiltration of land and is related to the pore state of the soil and the weight of the soil volume. The number and size of pores are determined by large pores. The more large pores, the greater the infiltration capacity. On the basis of the pore size, the clay fraction is rich in fine pores and poor in large and heavy pores, including low soil volume (Schoonover & Crim 2015). In contrast, the sand fraction contains large pores and slightly smooth pores, so the sand fraction infiltration capacity is greater than the clay fraction (Elfiati & Delvian 2010).

### Slope variables

Slope classification determines the amount of rainfall that becomes surface water and affects the discharge and volume of sub-watersheds (Mulia & Prasetyorini 2013). In addition to soil types, the slope is one of the main factors controlling surface flow and potential flooding (Wahid et al. 2016). The steeper the slope, the faster the water flowing in the watershed. Conversely, the gradient of the slope gets less steep, the flow of water in the watershed slows down so it is very possible for floods to occur.

The slope is based on the concept of the earth’s gravity. The steeper the slope, the weaker the gravitational force of the soil (Miscevic & Vlastelica 2014). On slopes that are too steep, the resultant force occurs because of the gravitational force with the ground shear force. The effect of slope on soil movement generally occurs in areas with slopes that are steeper. Areas that have the potential to cause flooding are upstream areas, because they have sharp and hilly slopes (Utama & Naumar 2015).

An assessment of the effect of slope variables in the Krueng Jreue Sub-Watershed was given a weight of 4. Harkat was dominated by a value of 20 at 29% and a value of 12 at 24%. The highest score is 20, the lowest score is 4 and the average score is 13.3.

Based on the four parameters of flooding, it can be seen that the key factors causing flooding in the study area are the biophysical conditions of the land, while other data such as extreme rainfall are a trigger for flooding. According to Riadi (2008), the land form is a representation of the shape of the earth’s surface. In connection with the risk and vulnerability of flood disasters, floodplain land forms have a close connection where the land form is a pool zone because of direct rainfall penetration or watershed that is unable to withstand upstream capacity.

The higher the total score, the more vulnerable the occurrence of floods. Most Land Mapping Units (LMU) in the Krueng Jreue Sub-Watershed have a high-level of vulnerability to flooding (moderate) with a total score ranging from 28 to 33 and averaging 30.0. The level of vulnerability of vulnerable floods has a total score ranging from 36 to 41, averaging 38.2.

### Flood vulnerability level based on land map unit

Classification of flood vulnerability based on the Krueng Jreue Sub-Watershed land map unit consists of four classes, namely: very vulnerable, vulnerable, fairly vulnerable (medium) and somewhat vulnerable. The classification of flood vulnerability based on the Krueng Jreue Sub-Watershed land map unit is listed in Table 6.

**TABLE 6:** Classification of flood vulnerability levels based on the land map unit.

Land mapping unit (LMU)	Land use	Large		Total score	Classification
		ha	%		
LMU 1	Open land	15.16	0.070	36.0	Vulnerable
LMU 2	Open land	4.90	0.020	29.0	Fairly vulnerable
Average TT	-	-	-	32.5	Vulnerable
LMU 3	Shrubs	313.40	1.350	38.0	Vulnerable
LMU 4	Shrubs	198.17	0.850	28.0	Fairly vulnerable
LMU 5	Shrubs	812.48	3.500	33.0	Fairly vulnerable
LMU 6	Shrubs	1735.24	7.470	29.0	Fairly vulnerable
LMU 7	Shrubs	858.38	3.700	22.0	Somewhat vulnerable
Average SB	-	-	-	30.0	Moderate
LMU 8	Meadow	658.18	2.830	31.0	Fairly vulnerable
LMU 9	Meadow	1860.95	8.020	29.0	Vulnerable
LMU 19	Meadow	1421.91	6.120	41.0	Vulnerable
LMU 20	Meadow	652.70	2.810	36.0	Vulnerable
LMU 21	Meadow	539.61	2.320	25.0	Somewhat vulnerable
Average PR	-	-	-	32.4	Moderate
LMU 10 (PM)	Settlement	103.88	0.450	42.0	Very vulnerable
LMU 11 (SW)	Rice fields	520.88	2.240	43.0	Very vulnerable
LMU 12	Moor	923.66	3.980	40.0	Vulnerable
LMU 13	Moor	0.56	0.002	33.0	Fairly vulnerable
Average TG	-	-	-	36.5	Vulnerable
LMU 14	Secondary forest	70.73	0.300	33.0	Fairly vulnerable
LMU 15	Secondary forest	285.03	1.230	29.0	Fairly vulnerable
LMU 16	Secondary forest	5046.18	21.730	28.0	Fairly vulnerable
LMU 17	Secondary forest	5601.00	24.120	18.0	Somewhat vulnerable
Average HS	-			27.0	Vulnerable
LMU 18 (HP)	Primary forest	1595.06	6.870	28.0	Fairly vulnerable

**Total**	**-**	**23 218.06**	**100.00**	**271.4**	**Fairly vulnerable**
**Average**	**-**	**-**	**-**	**33.9**	**Fairly vulnerable**

Results of analysing 21 land map units show that one unit map of class land is very vulnerable, six units of land map are vulnerable, 11 units of land map are fairly vulnerable and three units of land map are somewhat vulnerable to flooding. The highest total score is found in a land map unit of 11, which is 43 (paddy field); the lowest score in the land map unit is 17, which is 18, with a mean of 33.9 (fairly vulnerable).

### Flood vulnerability levels on some land use

The research area consists of eight types of land use, namely: open land, shrubs, grasslands, settlements, rice fields, moor, secondary forest and primary forest, with a total area of 23 218.06 hectares (ha). Flood vulnerability rates based on some land uses are listed in Table 7.

**TABLE 7:** Flood vulnerability levels on several land use types.

Land usage	Rainfall score	Land usage score	Soil infiltration score	Slope score	Total score	Classification
Open land	2.00	10	10.50	10	32.5	Fairly vulnerable
Shrubs	2.00	4	12.00	12	30.0	Fairly vulnerable
Meadows	2.00	10	6.00	12	30.0	Fairly vulnerable
Settlements	2.00	8	12.00	20	42.0	Vulnerable
Rice field	2.00	6	15.00	20	43.0	Very vulnerable
Tackles	2.00	6	10.50	18	36.5	Vulnerable
Secondary forest	2.25	2	9.75	13	27.0	Fairly vulnerable
Primary forest	2.00	2	12.00	12	28.0	Fairly vulnerable

**Total**	**16.25**	**48**	**87.75**	**117.00**	**269.00**	**-**
**Average**	**2.03**	**6**	**10.97**	**14.63**	**33.63**	**Fairly vulnerable**

Table 7 shows that paddy fields are particularly vulnerable to flooding because of relatively flat biophysical conditions (0% ≤ 8%) based on the Table. 4 and have clay texture classes. This resulted in a very slow infiltration rate of soil and soil compaction because of the conventional soil processing system. The decrease in soil infiltration power is one of the vulnerability factors to flooding (Rachmat & Pamungkas 2014) because the soil surface has degraded the ability to absorb rain water.

According to Munasinghe (1995) and Yoshida (2001), one of the indirect benefits of paddy fields is that they prevent flooding and erosion. Rice fields also have negative impacts on the environment, including the decline in the quality of paddy fields because of conventional farming practices. Physical damage to wetlands occurs because of poor management practices, such as a lack of rotation, continuous rice cultivation so that the soil is inundated throughout the year, shallow hacking using a rotary plow, that does not add organic matter or the return of plant residues to the soil, less soil puddling and the formation of shallow layers of tread.

According to Rahayu, Utami and Rayes (2014), morphologically, a wetland has a different horizon, colour and layers of plow tread. Differences in physical properties of paddy soil include structure, weight of soil volume and soil consistency as well as changes in the structure of granular soil until globs become massive on the coating. The higher soil volume and soil consistency in the plating layers have a stronger consistency than the dry soil.

### Zoning map of the flood vulnerability level

The highly vulnerable zone is a critical category of flood vulnerability caused by the gradient of a relatively flat slope, ranging from 0% ≤ 8% to form a landform unit of the land. The natural condition factors with the terrain form is one of the biophysical characteristics of vulnerable flooded land (Dewan 2015). According to Pratomo (2008), the slope influences the amount and speed of the surface flow. When the slope is shallow, the surface flow will be slower and the possibility of flooding becomes larger, while the steeper the slope, the surface flow will become faster and may not flood the area, so the negative impact and the risk of flooding become smaller.

The variable that causes this region to be particularly vulnerable is the infiltration rate that runs very slowly. Low infiltration rates reduce the amount of water stored in soil for plant growth which increases flooding and erosion caused by surface flows (Arsyad 2006). The slow rate of infiltration occurs, because the soil texture class is clay, with incomplete vegetation cover. Clay is a fraction of the soil consistancy quickly saturated with water in a wet state and has tight pores. If rain falls with intensity, the infiltration rate runs so slowly that it creates puddles on the surface. Rainfall is very influential in the research area.

In the northern and southern regions of the Krueng Jreue Sub-Watershed, the highest rainfall record is 270.5 and 249.2 mm month^−1^. The peak rainfall occurred between November and April despite a decrease in rain intensity in February, with a wet climate type. Surface flow can only be regulated by increasing the soil’s ability to store water, through increasing infiltration capacity, which is the maximum rate of water entering the soil (Kurnia et al. 2006). The amount of rain after infiltration is known, as more rain above the surface of the soil flows into a surface stream that causes flooding.

**FIGURE 1:**
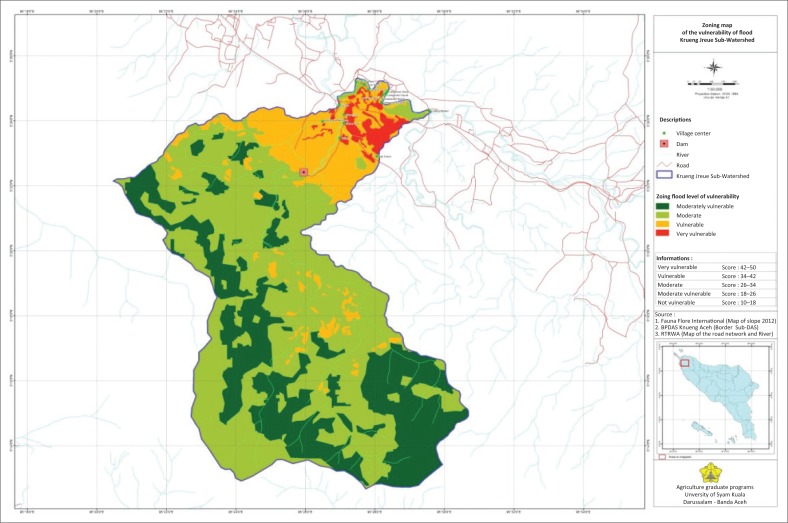
Zoning map of the vulnerability of flood in the Krueng Jreue Sub-Watershed.

High intensity rainfall causes floods. This is a characteristic of flooding in highly vulnerable areas. Areas that are particularly vulnerable and susceptible to flooding are the northern parts of the sub-watershed, which is along a downstream sub-watershed dominated by paddy fields, grasslands and shrubs. Floods occur from November to December, which are months with high rainfall. Four flood vulnerability zones are presented in Figure 1.

Flood vulnerability levels are: particularly vulnerable, vulnerable, moderately vulnerable and somewhat vulnerable, and the dominant ones are moderately vulnerable, somewhat vulnerable, vulnerable and highly vulnerable, with a mean total score of 30.0 each: 21.7, 38.8 and 43.0. The average flood vulnerability score was 33.38. The proportion of zones potentially vulnerable to flood disaster with a category of very vulnerable is 520.88 ha with a score of 43.0, the vulnerable area is 3117.31 ha has a score of 38.8, quite vulnerable is as wide as 12580.88 ha has a score of 30.0 and the somewhat vulnerable area of 6998.99 ha with score of 21.7, a broad proportion of each total area of research is 2.24%, 13.43%, 54.19% and 30.14% based on Table 9.

**TABLE 8:** Main and additional components of hydrological disaster vulnerability parameters.

No.	Parameter	Hydrological disaster vulnerability components	
		Main	Additional
1	Flood vulnerability level	Soil infiltration	Rainfall
		Slope	Air temperature
		-	Land use

**TABLE 9:** Classification of flood vulnerability levels based on the Krueng Jreue Sub-Watershed area.

Classification	Score	Large	
		ha	(%)
Very vulnerable	43.00	520.88	2.24
Vulnerable	38.80	3117.31	13.43
Quite vulnerable (moderate)	30.00	12580.88	54.19
Somewhat vulnerable	21.70	6998.99	30.14

**Total**	**133.50**	**23218.06**	**100.00**
**Mean**	**33.38**	**-**	**-**

#### Components and hydrological disaster mitigation efforts

Components that affect the level of vulnerability of hydrological disasters can be divided into two categories, namely the main components and additional components. The main components and additional vulnerabilities of hydrological disasters based on parameters of the level of flood vulnerability in the Krueng Jreue Sub-Watershed are listed in Table 8.

In above-normal rainfall conditions, the decrease in temperature and the physical condition of the slope < 3%, there is a threat of flood vulnerability disaster. The causes of flooding are not only hydro climatological factors, land use conditions, soil infiltration and physical potential hazards of water drainage and slope. The risk of catastrophic flooding is direct run-off in rainfall conditions above the normal projection period > 89 mm per month^−1^, which struck the slope < 3% in the face of various levels of related vulnerability (ICCSR 2010). Areas of significant risk or flood-prone areas are low-lying areas especially downstream of the watershed and rice paddies, with a flat slope. While the area that has the potential to cause flooding is the upstream sub-watershed, because it has a sharp and hilly terrain (Utama & Naumar 2015).

Differences in the degree of flood vulnerability in different land use in the Krueng Jreue Sub-Watershed are related to the magnitude of the risk of a hydrological disaster occurring. The lowest flood vulnerability (TKB) scores are found in primary and secondary forests with a low hydrological risk. While the highest TKB score is found in settlements, rice fields and moors with a high risk of hydrological disaster, so that a high-level hydrological disaster mitigation effort is needed. Primary and secondary forests in the Krueng Jreue Sub-Watershed have the lowest TKB, with a lower likelihood of floods and droughts.

To minimise the negative impacts and risks because of flood disaster in the Krueng Jreue Sub-Watershed, hydrological disaster mitigation is measured at the flood vulnerability zones with criteria of very vulnerable (score = 43.0) and vulnerable (score = 38.8). It is performed by technical means, namely the application of conservation principles through mechanical and vegetative means, as well as non-technical means through legal policy, law enforcement and socialisation in the form of sympathetic invitation to the community. It is in line with Salami et al. (2017) who suggest effective adaptation policies to minimise exposure and vulnerability to flood risk.

Mechanical (structural) disaster mitigation efforts are performed by increasing the water capacity of paddy fields and paddy systems. If this concept is adopted, downstream flooding can be suppressed, because most run-offs are accommodated in three places, namely paddy fields, terraced and hydrological networks on a level so that the water capacity of the Krueng Jreue Sub-Watershed can be improved, and only a small part of the water that runs downstream. Thus, the maximum discharge that occurs can be decreased and the response time can be extended (Nugroho 2015).

Furthermore, it is also important to optimise the development of the Krueng Jreue dam and the manufacture of flood retaining embankments to prevent the occurrence of river water flows, especially in vulnerable locations flooding the river. The Krueng Jreue dam was repaired by raising the embankment and maintaining the flood control building (Cury 1991).

To control flood disaster, it can also be managed by building dams. This aims to reduce the flood water level by improving river flow and channel normalisation. The shallow bottom of the river is deepened, while the river embankment on either side is widened. This method will improve the ability of the excess water reservoir and reduce the chances of overflowing water around the river. The dam is then able to keep surface flow reserves while removing them at manageable levels. To reduce sedimentation in the weir and siltation of the river, weir controls (check-dam) are made in the upper reaches of the river and areas susceptible to erosion. It aims to increase the filling of pores and percolation of soil by making rorak which will significantly decrease the outflow surface of the land parcels (Mogolion et al. 2016). This will certainly contribute to flood control.

Vegetal (non-structurally), disaster mitigation efforts are carried out by reducing surface flows into rivers and greening with hardwoods. The other efforts are by increasing water catchment and run-off control with agroforestry systems, planting crops which have deep rooting properties, which have firming roots and bind to soil aggregates and have a light biomass weight (Locatelli et al. 2015).

In a non-technical way, spatial planning is performed with an environmental perspective and the return of land function as its function, such as a forest is returned to a recharge area and a rice field area becomes a discharge area (Turner et al. 2000). Other efforts are controlled over spatial use through watershed management and flood areas, settlement system arrangement and early warning systems, licencing mechanisms to maintain ecosystem balance.

Another effort that needs to be performed in disaster mitigation is the mapping and incorporation of data of vulnerable elements with flood preparedness and mitigation activities. The next effort is mapping of flood vulnerability zones by using remote sensing techniques (Nastiti et al. 2015), monitoring and processing of biophysical land and meteorological data and making flood predictions using telemetry systems (remote observation and timely).

Another effort is the prohibition of land use for certain functions in the flooded areas. This is in line with the results of Doocy et al. (2013), which conclude that one of the hydrological disaster mitigation efforts is not to make the location of physical construction and settlement on river banks. Other non-technical efforts include socialisation of climate change and global warming to communities and stakeholders (Udmale et al. 2014) in the Krueng Jreue Sub-Watershed area.

## Conclusion

- The level of flood vulnerability consists of classes: (a) very vulnerable, an area of 520.88 ha; (b) vulnerable, covering an area of 3117.31 ha; (c) sufficiently vulnerable, an area of 12 580.88 ha; and (d) slightly vulnerable, covering 6998.99 ha, with each total score of 43.0, 38.8, 30.0 and 21.7.
- The level of flood vulnerability consists of: (a) very vulnerable (rice field) with a total score of 43.0; (b) vulnerable (settlement and moor), with a total score of 42.0 and 36.5, respectively; and (c) is vulnerable (open land, scrub, grassland, primary forest and secondary forest) with a total score of 32.5, 30.0, 30.0, 28.0 and 27.0.
- The main components affecting flood vulnerability are rainfall change, temperature rise and land use, while additional components are soil infiltration and slope.
- Mechanised hydrological disaster mitigation measures will be carried out by optimising the weir, embung, rorak and check-dam. Vegetative mitigation efforts are performed by reforestation and agroforestry systems. Non-technical, disaster mitigation efforts are pursued through legal policies, law enforcement, map-making, drought prediction and socialisation.
